# Upregulation of neuroreceptors on CD4+ and CD8+ T cells promotes their anti-tumor function

**DOI:** 10.1186/2051-1426-3-S2-P322

**Published:** 2015-11-04

**Authors:** Maria Teresa de Aquino, Thomas Hodo, Anil Shanker

**Affiliations:** 1 Meharry Medical College, Nashville, TN, USA; 2 Meharry Medical College School of Medicine / Vanderbilt-Ingram Cancer center, Nashville, TN, USA

## 

The nervous system can regulate the exacerbation of pro-inflammatory immune response predominantly in an immunosuppressive setting. Studies also suggest that glutamate, serotonin, dopamine, and substance P trigger immune responses such as cytokine secretion, integrin expression, and chemotaxis. We evaluated the role of neurotransmitters in modulating and/or activating T cells in mouse tumor models of kidney (RencaHA) and breast cancers (4T1HA) expressing viral hemagglutinin as a defined immunodominant antigen. We observed an expression of glutamate receptors GluR1, GluR3, GluR5, NMDA1 and NMDA2B as well as dopamine receptor DRD3, substance P receptor NK1, serotonin receptors 5HT7, and 5HT2B on mouse T lymphocytes. In particular, concomitant with the upregulation of T cell activation molecules CD25 and CD44, we found that both CD4^+^ and CD8^+^ T cells significantly upregulated expression of glutamate receptors (GluRs) following TCR stimulation, with CD8^+^ T cells maintaining a higher receptor expression for a longer time than that on CD4^+^ T cells. In mice bearing RencaHA and 4T1HA tumors also, HA-reactive CD8^+^ T cells in the tumor-draining LN and tumor-infiltrating lymphocytes showed an upregulated expression of GluR3 and GluR1 receptors. Moreover, proliferating CD8^+^ T cells presented higher levels of GluR3 and GluR1 when compared with non-proliferating T cells. We are currently investigating the dynamics of glutamate receptor signaling on various intracellular components of T cell activation signaling as well as cytokine expression, survival, and cytolytic function in the presence of glutamate receptor agonists and antagonists. Further, we observed that the expression of glutamate receptors could be modulated by cancer therapeutic proteasome inhibitor drug bortezomib concomitant with the expression of intracellular TCR signaling molecule CD3zeta and IFN-gamma in mice bearing RencaHA and 4T1HA tumors. Thus, pharmacological modulation of glutamate receptor signaling could be a novel strategy for enhancing anti-tumor immunity of T cells by overcoming tumor-induced immunosuppression, most likely by increasing T cell survival and cytolytic function. These findings shed new insights on unexplored neural-immune cross-talk mechanisms to overcome tumor immunosuppression and enhance anti-tumor T cell function with a potential to improve T cell immunotherapy of solid cancers.

